# Bridging the gap between adult and paediatric outcomes in HIV-1 vertically infected children: a single-centre comparison with adult data

**DOI:** 10.1111/j.1651-2227.2009.01440.x

**Published:** 2009-11

**Authors:** F Monpoux, P Puglièse, F Berthier, J Cottalorda, C Pradier

**Affiliations:** 1 Service de Pédiatrie, Hôpital de l’Archet II, Centre Hospitalier Universitaire de NiceNice Cedex, France; 2 Service des Maladies Infectieuses et tropicales, Hôpital de l’Archet II, Centre Hospitalier Universitaire de NiceNice Cedex, France; 3 Département d’information et informatique médicale, Hôpital de Cimiez, Centre Hospitalier Universitaire de NiceNice Cedex, France; 4 Laboratoire de virologie, Hôpital de l’Archet II, Centre Hospitalier Universitaire de NiceNice Cedex, France; 5 Département de santé publique, Hôpital de l’Archet II, Centre Hospitalier Universitaire de NiceNice Cedex, France

**Keywords:** Adult, Children, HAART, Prognosis, Viral load

## Abstract

Prognosis of HIV-1 infection dramatically improved during the last decade. Meanwhile, treatment-induced virological success has always been different in adult and children patients.

**Aim:**

To compare 10 years of follow up in HIV-1 vertically infected children and adult patients.

**Methods:**

Monocentric retrospective longitudinal analysis of vertically HIV-1-infected children and adult patients followed in the Nice University Hospital between 1999 and 2008. Immunological, virological and antiretroviral treatment data were recorded.

**Results:**

Forty children and 1752 adult patients were included. Between 1996 and 2008, the percentage of children receiving HAART increased from 3.2% to 91%. Mean CD4% in the paediatric group remained stable between 29 ± 8.1% in 1998 and 30 ± 9.4% in 2008. Mean adult CD4-cell count significantly increased from 410 in 1998 to 556 cells/mL in 2008. Logistic regression analysis showed that the children-to-adult difference for indetectability (HIV PCR-RNA below 400 copies/mL) was significant (p < 0.0001) with an odds ratio of 0.61 (CI^95th^: 0.52–0.72). Year-to-patient interaction was also significant with a decreasing divergence over time (p: 0.038).

**Conclusion:**

Nowadays as in adult patients, the control of HIV-1 replication is achieved in nearly eight of 10 children and the percentage of patients with severe immunodeficiency dramatically decreased compared with the mid 1990s.

## Introduction

During the mid 1990s, the development of highly active antiretroviral therapy (HAART) resulted in a significant decrease in morbidity and mortality among HIV-infected children (1). However, in most studies, the percentage of children achieving undetectable viral load was always lower than that observed among adult patients in controlled trials (1,2). The main given reasons were that young children tend to have higher viral loads and specific pharmacokinetics (2). Moreover, few licensed antiretroviral drugs were available as syrup or paediatric formulation and dosage.

Much progress has been made and pharmacokinetic studies in younger patients are now available for many drugs (3,4). Nowadays, the percentage of children achieving undetectable viral load while receiving HAART is expected to be similar to that observed among adult HIV-infected patients (5). To ascertain this assumption, we reviewed and compared our two local paediatric and adult databases.

## Subjects and Methods

Immunological, virological and antiretroviral treatment data from 40 HIV-1 vertically infected children (25 girls) followed between February 1995 and October 2008 were analyzed. The paediatric database was started prospectively in November 1991. For each consultation the child’s age, antiretroviral treatment, PCR-RNA HIV-1, CD4 and CD8 cell count and percentage were recorded. Children’s data were compared with those of adult patients recorded in the Nadis® database between 1998 and 2008. Nadis® is a computerized medical file for adult patients infected with HIV, HBV or HCV, initially activated in November 2000 in Nice and Toulouse. This database was previously described elsewhere (6). For all of the patients from both databases, all biological analyzes were performed in the same immunological and virological laboratories. Peripheral blood T-lymphocytes and subclass assays (CD4, CD8) were conducted by flow cytometry. Real-time plasma HIV RNA values were determined with the Cobas Amplicor® HIV-1 Monitor test (V.1.5) between 1995 and 2004, and with the Cobas Ampliprep®-Cobas TaqMan® HIV Monitor assay (Roche Diagnostics, Basel, Switzerland) afterwards, according to the manufacturer’s instructions.

To ensure cohesion during the 10-year period of follow-up, undetectable HIV-1 viraemia was defined as PCR-RNA below 400 copies per mL. All of the antiretroviral treatments were prescribed in agreement with the successive French recommendations [5). Two nucleoside reverse transcriptase inhibitors (NRTI) and one non-nucleoside reverse transcriptase inhibitor (NNRTI) or two NRTI and one protease inhibitor (PI) were considered as HAART for paediatric treatment. Definition of HAART was the same for adult patients apart from those compounds not yet currently available for children (fusion and integrase inhibitors).

All values are furnished as mean ± standard deviation. Statistical analysis was performed with SAS software version 9 (SAS Institute Inc., Cary, NC, USA) using a logistic regression analysis after adjustment for effect (patients * calendar years – Wald Chi-square test). The chi-square test was used for percentage comparisons (year-to-year comparison of percentages of adult and child patients with undetectable viral load).

## Results

Among the 1170 recorded episodes in the paediatric database, 832 were analyzed. Data from patients without treatment were excluded (n = 338). These mainly involved patients on structured treatment interruption or new patients before treatment initiation. Thus, between 1995 and 2008, for each patient, an average of 2.6 blood samples per year was analyzed (1.7 – 4.0). Mean follow up per patient was 88.7 ± 40.2 months (5.7–152.3).

**Figure 1:**
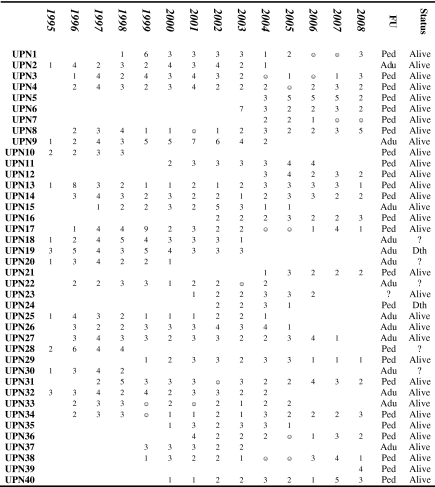
Children population and follow up. The number in each cell represents the total number of blood sample available for the patient during the year of follow-up Ped: follow up in the paediatric department, Adu: follow-up in the adult department, ?: lost to follow-up, Dth: death, 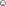: data excluded because of structured treatment interruption.

As expected, the mean age of the paediatric cohort increased slightly yearly during the observation period (Table 1). At the time of analysis, two patients had died from HIV infection, one was lost to follow up in 2005 and the remaining 37 patients were alive. Fourteen adolescents were transferred to the adult infectious diseases department between 2000 and 2008 after reaching the age of 18 years (Fig. 1). Clinical status according to the paediatric classification (7) was 17, 12 and 11 patients for stages N-A, B and C respectively. For the adult database, the number of included patients increased from 1154 to 1732 between 1998 and 2008 for a total count of 15 771 blood samples.

**Table 1:** Mean yearly age, immunological and virological parameters for the paediatric cohort

Years	Mean age (SD)	Mean CD4 (SD)	Mean CD4% (SD)	Mean log_10_ PCR-RNA (SD)
1995	8.2 (2.10)	303 (NA)	16.0 (NA)	4.95 (0.51)
1996	7.7 (2.90)	531 (18.04)	38.8 (18.04)	4.65 (0.83)
1997	8.2 (3.34)	596 (12.30)	22.6 (12.30)	4.24 (1.08)
1998	9.0 (3.22)	783 (8.13)	28.9 (8.13)	3.85 (1.27)
1999	9.3 (4.23)	878 (10.35)	29.2 (10.35)	3.78 (1.22)
2000	10.1 (4.37)	979 (10.80)	29.0 (10.80)	3.26 (1.11)
2001	10.3 (4.52)	934 (9.70)	30.1 (9.70)	2.86 (1.20)
2002	11.6 (4.66)	819 (9.59)	28.0 (9.59)	2.84 (1.27)
2003	10.7 (5.67)	943 (12.57)	31.1 (12.57)	2.6 (1.21)
2004	11.2 (4.72)	874 (9.39)	31.0 (9.39)	2.38 (1.10)
2005	10.6 (4.00)	890 (8.04)	34.2 (8.04)	2.42 (1.06)
2006	11.7 (3.68)	784 (10.55)	31.8 (10.55)	2.38 (0.95)
2007	12.6 (3.42)	701 (9.47)	31.1 (9.47)	1.95 (0.69)
2008	12.1 (4.60)	721 (9.42)	30.1 (9.42)	1.92 (0.56)
All	10.24 (4.37)	795 (10.32)	30.4 (10.32)	3.13 (1.37)

**Figure 2:**
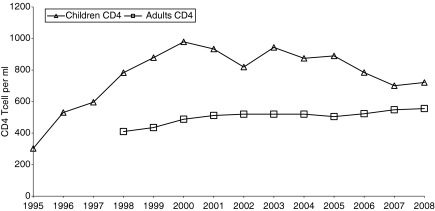
Mean yearly CD4 T-cell counts for adult patients (square) and infected children (triangle).

During the observation period, mean CD4% in the paediatric group remained stable between 29 ± 8.1% (783 ± 351 CD4 T-cells per mL) in 1998 and 30 ± 9.4% (721 ± 322 CD4 T-cells per mL) in 2008 (Table 1, Fig. 2). Meanwhile, the percentage of samples with severe CD4 T-cell deficiency (defined as below 15%) dropped from 20% in 1997 to 6.3% in 2007. During the same period, mean adult CD4 T-cell count increased significantly from 410 in 1998 to 556 cells/mL in 2008 (Fig. 2).

**Figure 3:**
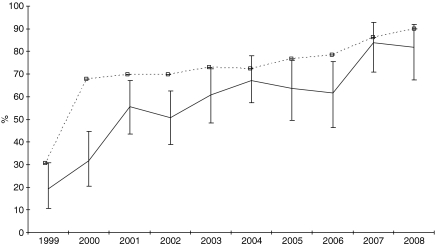
Yearly percentage of HIV-1 PCR-RNA <400 copies per mL in adult patients (dot curve) and HIV-1 vertically infected children (continuous curve with mean and 95th confidence interval).

**Figure 4:**
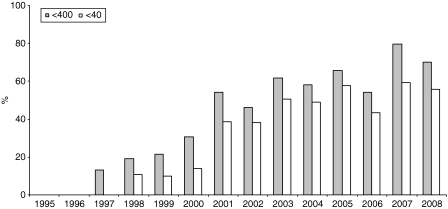
Yearly percentage of time with undetectable viral load for the entire paediatric cohort (grey square HIV-1 PCR-RNA <400 copies per mL, blank square HIV-1 PCR-RNA <40 copies per mL).

The mean yearly viral load measured in the paediatric cohort decreased dramatically from 4.65 ± 0.83 log_10_ (range: 4.0–5.67) in 1996 to 1.92 ± 0.56 log_10_ (range: 1.60–3.43) in 2008 (p < 0.001) (Table 1). Figure 3 displays the yearly percentage of samples with undetectable viraemia and compares adult and paediatric values. Chi square analysis showed that the difference was significant for the years 2000 (p < 0.001), 2002 (p < 0.005) and 2006 (p < 0.05). Logistic regression analysis showed that overall, the children-to-adult difference for indetectability was significant (p < 0.0001) with an odds ratio of 0.61 (CI95th: 0.52–0.72). Year-to-patient interaction was also significant with a decreasing divergence over time (Wald Chi-square; p = 0.038). Figure 4 displays the yearly percentage of time with undetectable viral loads for the entire cohort of patients. Thus, during the year 2007, our cohort of patients had an HIV-1 PCR-RNA below 400 copies per mL for nearly 80% of the time, assuming the fact that the blood samples obtained for each patient during the year reflected the true values of the year.

**Figure 5:**
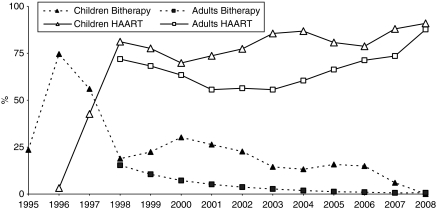
Yearly percentage of bi-therapy and highly active antiretroviral treatment (HAART) in HIV-1 vertically infected children (triangle) compared with adult patients (square).

Figure 5 shows treatment strategies provided to adult and paediatric patients. Between 1996 and 2008, the percentage of children receiving HAART increased from 3.2% to 91%. The percentage of PI used in the HAART combinations decreased from 100% in 1996 to 21% in 2001, then rose to 66% in 2008. NNRTI followed the opposite tendency (none in 1996; 53% in 2001; 39% in 2008). During the same period, dual NRTI combination was reduced from 75% to none of the infected children in 2008.

## Discussion

Prospective studies on efficacy, tolerability and safety of new antiretroviral drugs remain essential to explore and provide new tools against HIV infection in children. Most of the HAART-based combinations can achieve sustained viral suppression and beneficial clinical and immunological responses. Meanwhile, when considering a given population living with AIDS, cross-sectional analysis of viral suppression showed results far from those obtained in prospective controlled studies (8–10). Indeed, tolerability and adherence to treatment may vary in great proportion resulting in a heterogeneous cohort of patients. Morever, in paediatric patients, adherence to continuous oral therapy is not self-directed but usually depends on parental contribution and conviction. Specific attention should also be addressed to HIV-1 chronically infected adolescent regarding to adherence as mentioned in most recommendations (5,9).

Until now, in most phase I/II trials of new antiretroviral drugs, the percentage of children with undetectable viral load was lower than observed in adults, which is a similar trend to that observed in young outpatients in clinical practice. In 2000, the Italian registry showed a major improvement in the survival of perinatally HIV-infected children as a result of the introduction of combined antiretroviral therapies since 1996. This population-based multicentre observational study was the first paediatric study to provide information on clinical effectiveness at the population level (11).

More recently, the study published in 2004 by the Collaborative HIV Paediatric Study group from UK and Ireland (CHIPS) confirms that there are critical differences in the predictors of immunological and virological response to HAART in previously untreated children and adults. Overall, 60% of their children achieved HIV-1 RNA levels below 400 copies/mL within the first 6 months of treatment, ranging from 48% in the youngest (<2 years old) to 76% in the oldest patients. No calendar year of starting HAART effect was noted (12). Conversely, in a recent update of these cohorts, only calendar time predicted virological response in multivariate analysis. For the authors, this discrepancy is mainly due to a better understanding of HAART management together with improved formulations and simpler regimen (13).

Our data confirm that henceforward, in industrialized countries, as is the case with adult patients, most of the children receiving HAART for HIV infection achieved control of viral replication. We observed that in <3 years between 1999 and 2001, the percentage of HIV PCR-RNA <400 copies per mL increased dramatically in adults and children from 20–30% to nearly 60–70%. Progression among children was delayed compared with the situation observed among adults. There are probably at least two main reasons for this trend: first, the later availability of paediatric drug formulations for the new antiretroviral treatments, second, the need for specific paediatric data for pharmacokinetics. Variation in immunological status during the observation period was less obvious. We mainly observed a marked decrease in the percentage of children with the most severe immunodeficiency (<15% CD4). In both our adult and paediatric cohort, yearly evaluation of immune status showed a trend towards improvement.

Our study is quite different from the COHERE (Collaboration of Observational HIV Epidemiological Research Europe) study that addressed treatment outcome by age in 49 921 patients ranging from 4 days to 87 years across 30 European countries. Better virological but poorer immunological responses in older individuals were observed. Meanwhile, as in our study, the chance of experiencing a good virological response was also associated with calendar period (8).

We must, however, acknowledge the fact that our study has a number of limitations. Namely, the small number of children, our definition of undetectable viral load now being replaced by ultrasensitive assays with lower quantification limits (<40–50 copies/mL) and unknown or unmentioned confounders not registered in our databases (B and C hepatitis status, gender, route of transmission in the adult database …) must probably be considered.

Morbidity and mortality rates among perinatally HIV-1-infected children continue to decrease over time as recently confirmed in the group from UK and Ireland (12). Meanwhile, nearly 40% of the children aged 15 years and above have been exposed to drugs from each of the three main HAART classes (12). Resistance analysis from the same cohort published elsewhere showed that only 14% of the 166 children with a resistance test had wild-type virus (13). These data underline the need for real-time and continuous monitoring of paediatric cohorts to ensure an optimal transition from paediatric to adult department. It is therefore important to consider that, as recently published in a multicohort study, the average number of years remaining to be lived at the age of 20 dramatically increased since 1996 but remains about two-thirds of that in the general population of high-income countries (14).

## Conclusion

HAART is now recommended as standard treatment regimen in both adult and paediatric HIV-infected patients living in industrialized countries (5,9). Analysis of our cohort showed that nowadays the control of HIV-1 replication is achieved in nearly eight of 10 children and that the percentage of patients with severe immunodeficiency globally dramatically decreased compared with that of mid 1990s.

## Abbreviations

HAART: Highly active antiretroviral treatment
NNRTI: Non-nucleoside reverse transcriptase inhibitor
NRTI: Nucleoside reverse transcriptase inhibitor
PI: Protease inhibitor
